# Development of a Multivalent Subunit Vaccine against Tularemia Using Tobacco Mosaic Virus (TMV) Based Delivery System

**DOI:** 10.1371/journal.pone.0130858

**Published:** 2015-06-22

**Authors:** Sukalyani Banik, Ahd Ahmed Mansour, Ragavan Varadharajan Suresh, Sherri Wykoff-Clary, Meenakshi Malik, Alison A. McCormick, Chandra Shekhar Bakshi

**Affiliations:** 1 Department of Microbiology and Immunology, New York Medical College, Valhalla, New York, United States of America; 2 College of Pharmacy, Touro University California, Mare Island, Vallejo, California, United States of America; 3 Albany College of Pharmacy and Health Sciences, Albany, New York, United States of America; Midwestern University, UNITED STATES

## Abstract

*Francisella tularensis *is a facultative intracellular pathogen, and is the causative agent of a fatal human disease known as tularemia. *F*. *tularensis* is classified as a Category A Biothreat agent by the CDC based on its use in bioweapon programs by several countries in the past and its potential to be used as an agent of bioterrorism. No licensed vaccine is currently available for prevention of tularemia. In this study, we used a novel approach for development of a multivalent subunit vaccine against tularemia by using an efficient tobacco mosaic virus (TMV) based delivery platform. The multivalent subunit vaccine was formulated to contain a combination of *F*. *tularensis* protective antigens: OmpA-like protein (OmpA), chaperone protein DnaK and lipoprotein Tul4 from the highly virulent *F*. *tularensis *SchuS4 strain. Two different vaccine formulations and immunization schedules were used. The immunized mice were challenged with lethal (10xLD_100_) doses of *F*. *tularensis *LVS on day 28 of the primary immunization and observed daily for morbidity and mortality. Results from this study demonstrate that TMV can be used as a carrier for effective delivery of multiple *F*. *tularensis *antigens. TMV-conjugate vaccine formulations are safe and multiple doses can be administered without causing any adverse reactions in immunized mice. Immunization with TMV-conjugated *F*. *tularensis *proteins induced a strong humoral immune response and protected mice against respiratory challenges with very high doses of *F*. *tularensis* LVS. This study provides a proof-of-concept that TMV can serve as a suitable platform for simultaneous delivery of multiple protective antigens of *F*. *tularensis*. Refinement of vaccine formulations coupled with TMV-targeting strategies developed in this study will provide a platform for development of an effective tularemia subunit vaccine as well as a vaccination approach that may broadly be applicable to many other bacterial pathogens.

## Introduction

*Francisella tularensis* is the causative agent of a fatal human disease known as tularemia [1,2]. *Francisella* is divided into two species; *tularensis* and *philomiragia* [3,4]. There are four subspecies of *F*. *tularensis*: *tularensis* (also known as *F*. *tularensis* type A), *novicida*, *mediasiatica* and *holarctica* (also known as *F*. *tularensis* type B) [5]. Of these, only *F*. *tularensis* subsp. *tularensis* and subsp. *holarctica* can cause disease in immunocompetent humans. *F*. *tularensis* SchuS4 strain is a representative of *F*. *tularensis* subspecies *tularensis*. *F tularensis* has been classified as Tier 1 Category A Select Agent, which is the highest priority category on the list of CDC among other potential Biothreat agents [6]. The bioweapon potential of *F*. *tularensis* is on account of its extreme virulence, low infectious dose, ease of aerosol dissemination and capacity to cause severe illness and death in a very short period of time [7]. No licensed vaccine is currently available in the USA for prevention of tularemia [8,9]. Considering the bioweapon potential of *F*. *tularensis* and repercussions of 2001 anthrax attack in the USA, there has been an increased interest in development of vaccine and effective countermeasures against bioterror agents. An ideal solution for prevention of tularemia occurring naturally or consequent to the use of *Francisella* as a bioweapon or an act of bioterrorism is to develop a safe and effective vaccine capable of inducing long lasting protection in a relatively short period of time [10].

In the last 100 years since the discovery of *F*. *tularensis*, three broad approaches comprising of killed whole cell, live attenuated and subunit vaccines have been employed for vaccine development, but none of these have been successful [11]. Although, a Live Vaccine Strain (LVS) developed from the Russian strain *F*. *holarctica* S15 is protective, it retains residual virulence in humans when immunized via aerosol or intranasal (i.n.) routes. Due to adverse reactions and residual virulence, LVS is not approved by the FDA for mass immunizations in the USA. Attenuated mutants of *F*. *tularensis* SchuS4 or the LVS containing single gene deletions have shown better protective efficacy in mouse models of tularemia [12,13,14,15,16,17]. However, these mutants pose a potential possibility of reversion to fully virulent forms. Inactivated LVS or SchuS4 tularemia vaccines have demonstrated poor protective efficacies against challenges with virulent *F*. *tularensis* [11,18,19,20]. Several efforts to develop subunit tularemia vaccine have met with limited success. The primary shortcomings have been the constituents of subunit vaccines which contained either a single surface associated antigenic component of *F*. *tularensis* such as LPS or specific immunoreactive proteins such as GroEL, DnaK, FopA, KatG or a *F*. *tularensis* specific lipoprotein Tul4 [21,22,23,24,25,26,27,28]. Despite being immunogenic, these single subunit vaccines failed to provide protection against virulent *F*. *tularensis* strains. The possible explanations for their failure could be that single proteins are not sufficient or that the vaccine formulations lacked right combination of antigens required for induction of a protective immune response. The challenges thus far in development of multivalent subunit vaccines have been the availability of suitable approaches for consistent preparation and efficient delivery of multiple antigens through mucosal routes.

The goal of this study was to explore vaccine potential and preclinical development of a multivalent subunit vaccine against tularemia using an efficient TMV based delivery platform. The premise behind utilizing a novel TMV-conjugated vaccination strategy is founded on the proven efficacy of TMV vaccines in stimulating robust humoral and cellular immune response without the requirement of an additional adjuvant [29]. TMV as an antigen carrier provides two important functions: **1)** because of the virus architecture and size, TMV provides for active and robust uptake by dendritic cell and activation of key surface markers *in vitro* and *in vivo* resulting in effective antigen presentation [30,31]. **2)**, TMV provides adjuvant effects, either because of the repetitive antigen display that mimics virus surfaces which is important for generation of potent antibody responses, or because of the presence of virus RNA (albeit non-functional) which is important for inducing cell mediated immunity (or both). Conjugating an immunogenic subunit vaccine protein to the surface of TMV promotes antigen uptake and improves an antiviral response against the subunit protein. A recent study demonstrated single dose potency of a TMV-hemagglutinin (TMV-HA) vaccine in an influenza challenge model without the need for an adjuvant [32]. Because TMV is not a human pathogen [33], TMV is inherently safe. In addition, TMV does not show evidence of neutralizing antibodies in individuals, so it can be used repeatedly for boosting [31,32]. These characteristics of TMV are extremely important in producing a safe, effective vaccine that can stimulate protection against *F*. *tularensis* challenge.

We investigated the vaccine potential of a multivalent tularemia vaccine by chemically conjugating TMV to multiple protective antigens of *F*. *tularensis*. We used purified recombinant proteins DnaK (*FTT1269c*), OmpA (*FTT0831c*) and Tul4 (*FTT0901*) of *F*. *tularensis* SchuS4 and determined the vaccine potential of TMV-*F*. *tularensis* protein conjugate vaccine. When used in vaccine formulations, both DnaK and Tul4 have been shown to render some degree of protection against *F*. *tularensis* LVS in vaccinated mice [34,35]. This was the rationale for inclusion of these proteins in our studies to investigate the efficacy of TMV-conjugate vaccine. In addition to DnaK and Tul4, we also included OmpA-like protein in the conjugate cocktail based on its surface exposed structures, role in innate immune subversion both *in vitro* and *in vivo* [13,36,37] and exclusive reactivity of this protein with the serum from successfully vaccinated individuals as well as mice [38,39,40,41]. This study demonstrates that TMV effectively delivers multiple *F*. *tularensis* antigens to induce protective immune responses in mouse model of respiratory tularemia and provide a proof-of-concept for the feasibility of TMV as a carrier for bacterial antigenic proteins.

## Materials and Methods

### Ethics Statement

This study was carried out in strict accordance with the recommendations and guidelines of National Council for Research (NCR) for care and use of animals. All the animal experiments were conducted in the centralized Animal Resources Facilities of Albany Medical College and New York Medical College licensed by the USDA and the NYS Department of Health, Division of Laboratories and Research and accredited by the American Association for the Accreditation of Laboratory Care. The use of animals and protocols were approved by the Institutional Animal Care and Use Committee (IACUC) of New York Medical College (Protocol Number 30-2-0414H). Mice were administered an anesthetic cocktail consisting of ketamine (5 mg/kg) and xylazine (4 mg/kg) and underwent experimental manipulation only after they failed to exhibit a toe pinch reflex. Mice exhibiting more than 20% weight loss, anorexia, dehydration and impairment of mobility were removed from the study and euthanized by approved means. Humane endpoints were also necessary for mice which survived at the conclusion of the experiment. Mice were administered an anesthetic cocktail of ketamine and xylazine intraperitoneally and then euthanized via cervical dislocation followed by cardiac puncture, a method that is consistent with recommendations of the Panel on Euthanasia of the American Veterinary Medical Association. In all experimental procedures, efforts were made to minimize pain and suffering.

### Bacterial Strains

*F*. *tularensis* LVS (American Type Culture, ATCC 29684; Rockville, MD) used in this study was obtained from BEI Resources, Manassas, VA. *F*. *tularensis* LVS was grown on Mueller-Hinton (MH) chocolate agar plates (BD Biosciences, San Jose, CA) or MH-broth (MHB; BD Biosciences, San Jose, CA) supplemented with 0.021% w/v, Anhydrous Calcium chloride, 0.000138% w/v Hydrous Magnesium Chloride, 0.00021% w/v 10% Glucose, 10% v/v, 2.5% Ferric Pyrophosphate, 2.5% v/v Isovitalex (BD Biosciences, San Jose, CA). The active mid-log phase bacteria grown in MHB were harvested, aliquoted into sterile 1.5 mL cryovials and stored at −80°C for further use.

### Expression and Purification of Recombinant Proteins

The *dnaK* gene (*FTT1269c*) of *F*. *tularensis* SchuS4 cloned in *E*. *coli* expression vector pDSET17 was obtained from Harvard Institute of Proteomics. The genes of *F*. *tularensis* SchuS4 encoding for OmpA (*FTT0831c*) and Tul4 (*FTT0901*) were cloned into the pPROEX Htb vector (Invitrogen). The plasmids were transformed into *E*. *coli* (BL21/XL10 Gold) strain, induced for expression by IPTG, and purified by metal affinity chromatography. The purity of the proteins was confirmed by SDS-PAGE and western blot analysis using anti-6His antibodies.

### Purification of TMV-Lysine Virus Particles

TMV was genetically engineered to express coat protein containing a surface exposed lysine [42]. Infectious TMV RNA was inoculated onto 30 day *Nicotiana benthamiana* plants, and harvested for virus 10 days later according to previously described protocols [42,43]. Briefly, plant tissue was homogenized in 0.86M NaCl, 0.04% w/v sodium metabisulfite (0.5 g of tissue/ml of buffer), adjusted to pH 5.0, heated to 47°C for 5 min, and then chilled to 4°C. Homogenate was centrifuged at 6000 × g for 20 min, and then the clarified supernatant was precipitated with 5% Poly Ethylene Glycol (PEG) 8000 at 4°C, and spun at 12,000 × g for 10 min at 4°C to recover the virus. PEG pellets were resuspended in PBS, and re-precipitated with PEG a second time. Final PEG pellets were resuspended in PBS at 1:10th homogenization volume, and final protein concentration was measured by Bicinchoninic Acid (BCA). Purity was determined (typically >98%) by SDS-PAGE.

### Conjugation of *F*. *tularensis* Proteins to TMV

In order to use TMV as a platform, recombinant *F*. *tularensis* proteins were chemically conjugated to decorate the surface of TMV. For conjugation reaction, purified–TMV-Lysine and individual purified recombinant proteins OmpA, DnaK, and Tul4 were mixed at 1:1 molar ratios. The conjugation reaction was carried out by adding 5mM of 1- Ethyl-3-(3-dimethylaminopropyl) carbodiimide (EDC) and 1mM of N-hydroxysuccinimide (NHS). The conjugation mixture was then incubated for various time intervals to achieve maximum conjugation efficiency. The reaction time that generated the least amount of free protein (2 hours) was used in scale up conjugation reactions (5mg antigen with 5mg TMV) for vaccine potency testing. For the TMV monoconjugate vaccine, where all three proteins were reacted together onto the same virus, each protein was mixed with TMV at a 30% molar ratio (1x TMV, 0.3x each protein) and reacted for 2 hours to ensure complete conjugation. The efficiency and successful conjugation of recombinant proteins of *F*. *tularensis* SchuS4 to the TMV virion was determined by 8–16% Tris-Glycine SDS-PAGE.

### Safety, Immunogenicity and Protective Efficacy of TMV-Conjugate Vaccines

#### Mice

Wild-type C57BL/6 mice were purchased from Charles River Laboratories, NY. Six to eight weeks old female mice were used in all experiments. All mice were maintained in environmentally controlled and pathogen-free animal facility of New York Medical College. All mice that were to be immunized or challenged were anesthetized by i. p. injection of a cocktail of Ketamine and xylazine to facilitate delivery of the inoculum to the respiratory compartment. All mice experiments were performed according to the guidelines and protocols approved by the IACUC at New York Medical College.

#### Vaccine formulations

**Fig 1:**
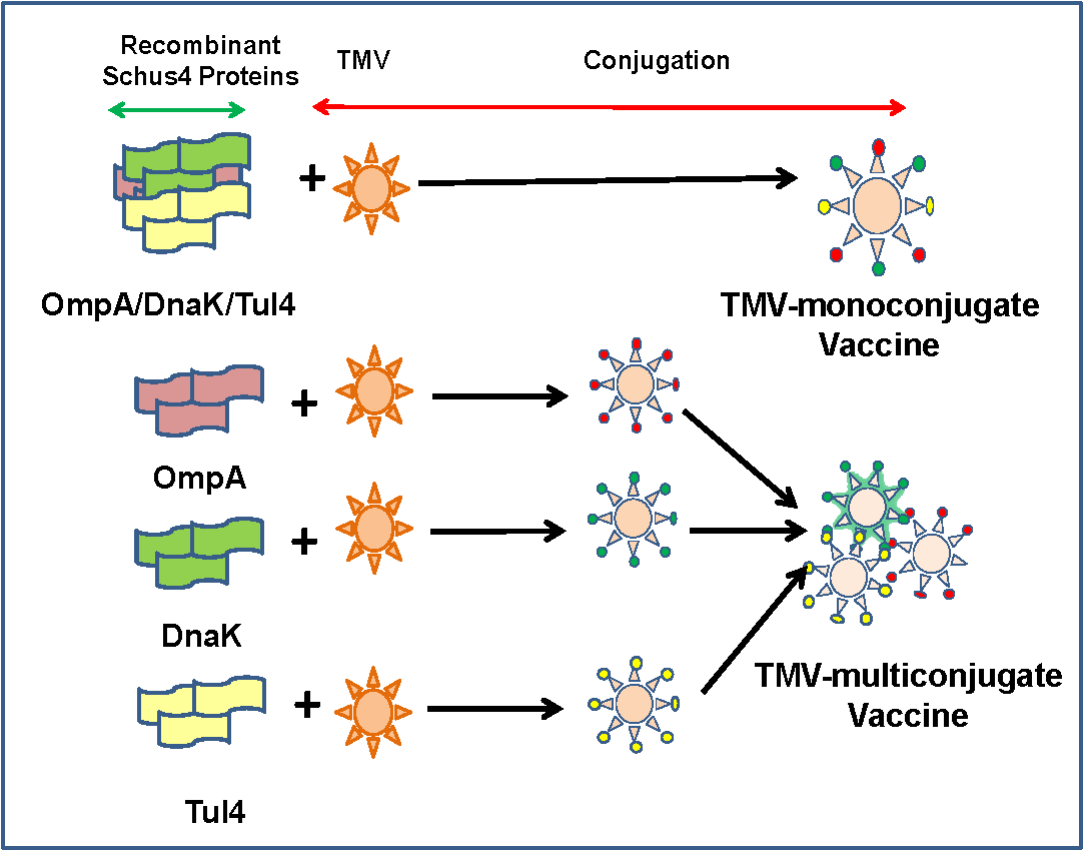
Vaccine Formulations. Two different vaccine formulations were used. In the first vaccine formulation all three recombinant proteins OmpA, DnaK and Tul4 were conjugated to a single TMV virion (TMV-monoconjugate vaccine). The second vaccine formulation contained each recombinant protein of *F*. *tularensis* conjugated individually to TMV and then mixed in equal concentrations to generate a TMV-multiconjugate vaccine.

Two different vaccine formulations were used. In the first formulation, all three recombinant proteins OmpA, DnaK and Tul4 were conjugated to a single TMV virion. This vaccine formulation was designated as TMV-monoconjugate vaccine. Mice were immunized with 60μg of TMV monoconjugate vaccine (~30μg TMV and 30μg of recombinant proteins). In the second vaccine formulation, each individual protein was conjugated to the TMV individually (10μg TMV + 10μg recombinant protein) and then each of the three TMV-protein conjugates were mixed in equal concentrations [20μg x 3 = 60μg (30μg TMV + 30μg recombinant proteins)]. This formulation was designated as TMV-multiconjugate vaccine (Fig 1). Based on the amount of TMV that each of the vaccinated mouse received (30μg), mice inoculated with an 30μg of TMV served as controls.

#### Immunization schedules

**Fig 2:**
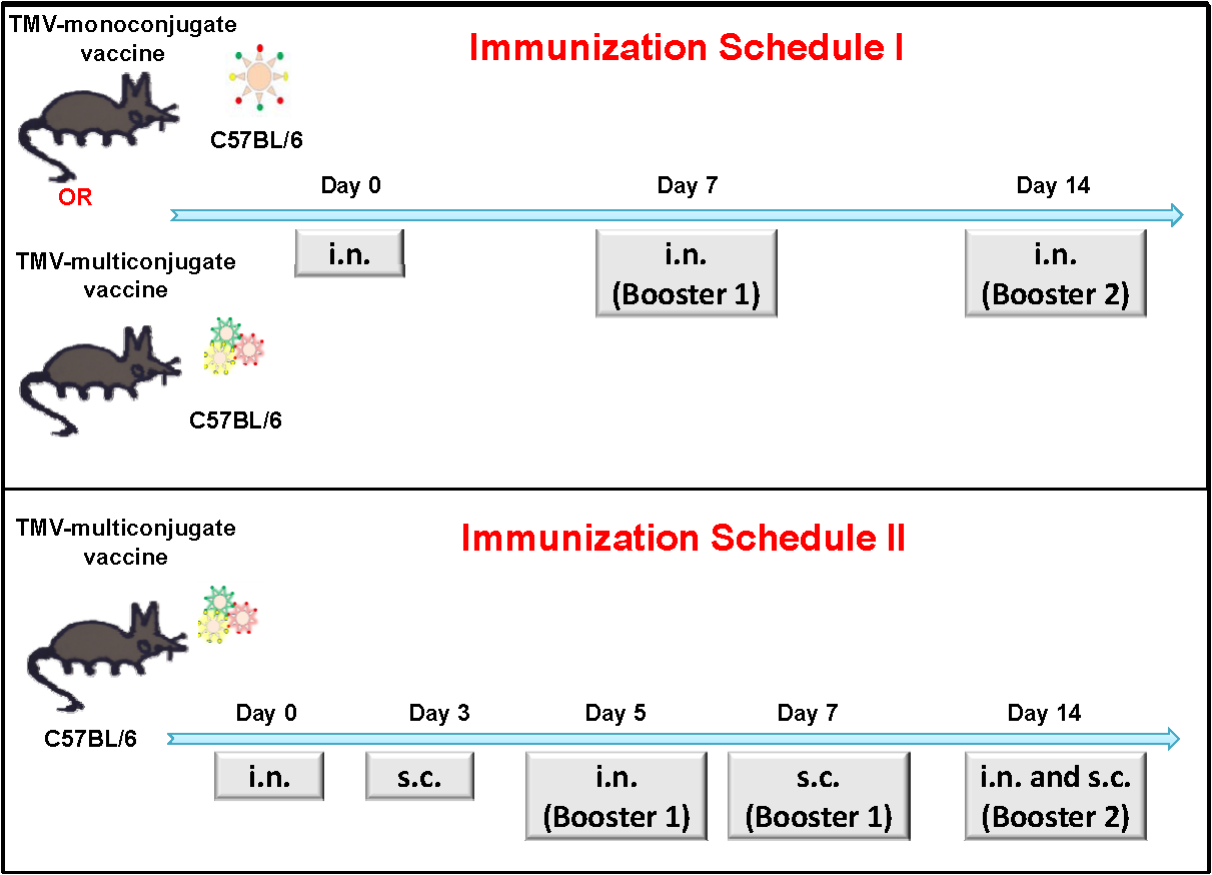
Immunization Schedules I and II. **(A)** C57BL/6 mice were immunized intranasally (i.n.) either with TMV-monoconjugate (60 μg/mouse) or TMV-multiconjugate vaccine formulations (20 μg each of OmpA-TMV; DnaK-TMV and Tul4-TMV conjugates. Total 60 μg/mouse) and booster vaccinations were administered i.n. using dosages similar to those for primary immunization on days 7 and 14 of the post-primary immunization (Schedule I). **(B)** Alternatively, mice were administered TMV-multiconjugate vaccines with booster immunizations i.n. on day 5 and 14 and subcutaneously (s.c.) on days 7 and 14 post-primary immunization (Schedule II). The dosages used were similar to those described for TMV-multiconjugate vaccine in **A**. Mice inoculated with TMV (30 μg/mouse) in a manner similar to the vaccinated groups were kept as controls.

Two different immunization schedules were used. In the first immunization schedule (Schedule I) C57BL/6 mice were immunized intranasally (i.n.) with 60μg of TMV monoconjugate or TMV-multiconjugate vaccine. Mice were immunized i.n. with 30μl (15μl/ nostril) volume of each of the vaccine formulation or the TMV controls. Booster vaccinations using dosages similar to the primary immunization were administered on days 7 and 14 after the primary immunization. Mice receiving 30μg of TMV alone and administered in a fashion similar to the vaccine groups were kept as controls (Fig 2A). Mice were monitored for any adverse reaction following each vaccine administration.

Mice immunized employing schedule II were vaccinated only with TMV-multiconjugate vaccine. C57BL/6 mice were immunized i.n. on day 0, 5 and 14 with a total of 60μg of TMV-multiconjugate vaccine. These mice also received similar vaccination dose of TMV-multiconjugate vaccine subcutaneously (s.c.) on days 3, 7 and 14. Control mice received 30 μg of TMV following the immunization schedule identical to those for the TMV-multiconjugate vaccine group (Fig 2B). All vaccinated and control mice were monitored for any adverse reaction following each primary and booster immunizations. All the vaccinated mice were bled periodically to collect serum to determine antibody responses. The Schedule I immunizations were based on a number of vaccination studies using inactivated or subunit vaccines of *F*. *tularensis* [18,34,44]. The aggressive immunization strategy (Schedule II) was based on our recent report indicating that alternate i.n. and s.c. booster immunizations enhanced protection in immunized mice against an i.n. challenge with *F*. *tularensis* SchuS4 strain [45] and the vaccination schedule recommended for commercially available oral typhoid vaccine, Vivotif.

#### Determination of antibody responses in immunized mice

For determination of anti—*F*. *tularensis* antibody levels in vaccinated mice following the immunization schedules described above, ELISA was performed using lysates made from *F*. *tularensis* SchuS4 or LVS strains. The formalin fixed SchuS4 was obtained from BEI Resources, Manassas, VA. For ELISA 96-well microtiter plates were coated with 1×10^7^ CFU/ml of either *F*. *tularensis* SchuS4 or LVS in bicarbonate buffer. *F*. *tularensis*-specific antibody levels for total IgG, IgG1, IgG2a and IgG2b in serum samples collected from immunized mice on 28 post-immunization were determined by ELISA. Serum collected from naïve mice or mice that received TMV were used as controls. To determine the level of antibodies induced against each individual protein of the TMV-monoconjugate or the TMV-multiconjugate vaccine each individual ELISA was performed by coating plates with 1μg of each individual purified recombinant OmpA, DnaK and Tul4 proteins. The protein specific total IgG levels were determined in serum from vaccinated mice collected on day 28 post-immunization. Antibody titers were calculated from linear regression curves as the inverse of the serum dilution that showed an OD_450_ value 2.5 times above the controls, and expressed as Log_10_ values.

To determine if antibodies generated in vaccinated mice are capable of identifying native and recombinant *Francisella* OmpA, DnaK and Tul4 proteins, western blot analysis was performed. Serum collected on day 28 from mice immunized with TMV-multiconjugate vaccine utilizing Schedule II was used for western blot analysis. Eight micrograms each of *F*. *tularensis* LVS and SchuS4 lysates were resolved on SDS-PAGE, transferred to nitrocellulose membrane and blotted against pooled serum from immunized mice. Serum collected from mice immunized with TMV was used as a control. To determine if antibodies from immunized mice reacted with recombinant proteins as well, 1μg of purified recombinant proteins were used in western blot analysis.

#### Challenge studies

To determine the protective efficacy of TMV-vaccine against a high challenge dose of LVS, all immunized mice were challenged i.n. with 10×LD_100_ (1×10^5^ CFU) dose of *F*.*tularensis* LVS on day 28 of the primary immunization. The actual numbers of *F*. *tularensis* inoculated into mice at that time of challenge were confirmed by plating serial dilutions on MH-chocolate agar plates and counting the colonies 48 hours later.

#### Post-challenge studies

All the challenged mice were observed daily for signs of morbidity and/or mortality for a period of 21 days. To monitor the progression of infection all challenged mice were weighed every day until they regained their original body weight.

### Statistical Analysis

All data for antibody levels of immunized mice were statistically analyzed using InStat program (Graph-Pad Software). The results were expressed as Means ± S.D. The survivals data were expressed as Kaplan-Meier survival curves and statistical significance for survival results were evaluated by analyzing the mean time to death by the Log-Rank test.

## Results

### Purification, and Conjugation of DnaK, OmpA and Tul4 Proteins of *F*. *tularensis* SchuS4 to TMV

**Fig 3:**
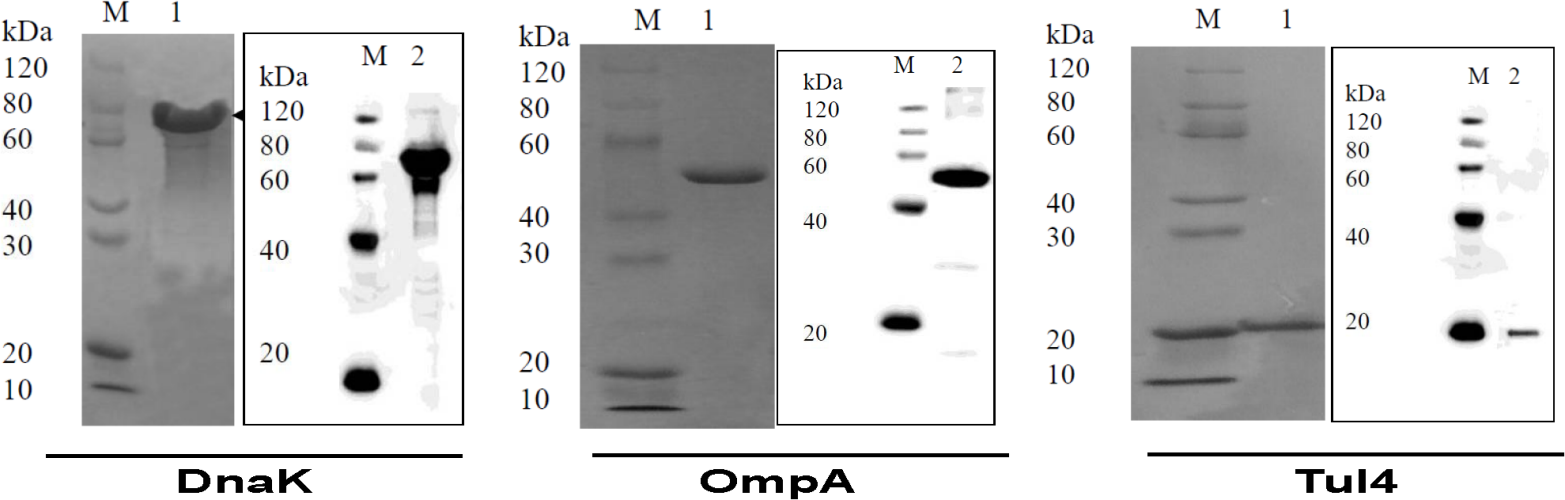
Expression and Purification of Recombinant DnaK, OmpA and Tul4 Proteins of *F*. *tularensis* SchuS4. Purification of recombinant OmpA, DnaK and Tul4 proteins of *F*. *tularensis* SchuS4 proteins was confirmed by SDS-PAGE and western blot analysis using anti-His antibodies.

**Fig 4:**
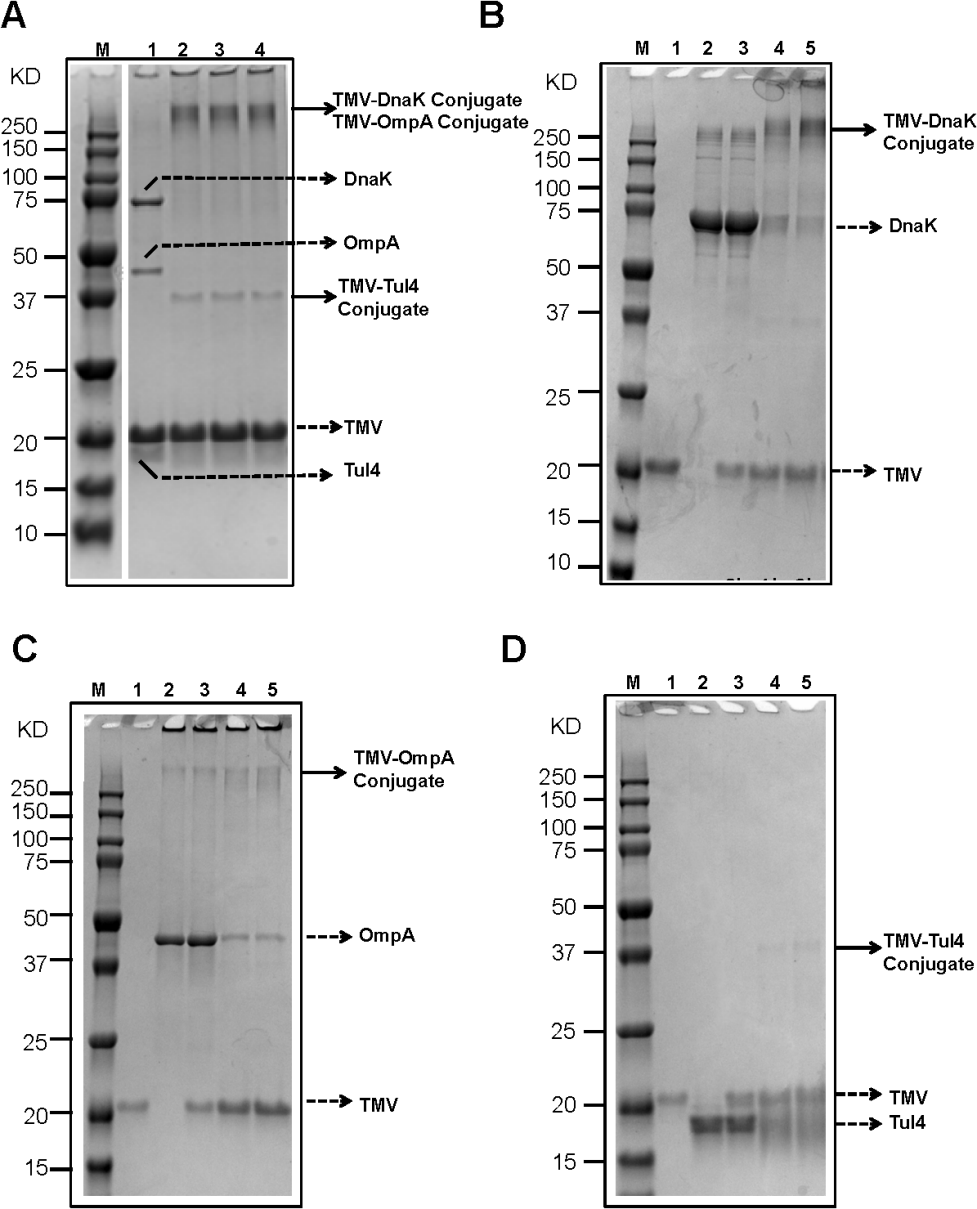
Conjugation of DnaK, OmpA and Tul4 Proteins of *F*. *tularensis* SchuS4 to TMV. Purified OmpA, DnaK and Tul4 proteins were combined with purified TMV and incubated with EDC and NHS for 0, 30 min, 1, or 2 hours as described in Methods section. Two μg of TMV or recombinant proteins DnaK, OmpA, Tul4 or 4 μg of the TMV-protein mixtures were resolved on an 8–16% SDS-PAGE gel to observe conjugation products indicated by changes in the molecular masses of the starting materials. **(A)** Conjugation of DnaK, OmpA and Tul4 to a single TMV virion to generate TMV-monoconjugate vaccine. The progress of conjugation process was observed over a period of time: Lane M = Precision Plus Dual Color standard (BioRad) Marker; Lane 1 = TMV-protein mix, 0 min; Lane 2 = TMV-protein mix, 30 min; Lane 3 = TMV-protein mix,1 hour; Lane 4 = TMV-protein mix, 2 hours. **(B, C, D)** Kinetics of DnaK, OmpA and Tul4 TMV-protein conjugations over a two hour incubation period to generate TMV-protein conjugates. The individual TMV-protein conjugates were then admixed to generate TMV-multiconjugate vaccine. Lane M = Precision Plus Dual Color standard (BioRad) Marker; Lane 1 = TMV; Lane 2 = Recombinant protein; Lane 3 = TMV-protein mix, 0 hour; Lane 4 = TMV-protein mix, 1 hour; Lane 5 = TMV-protein mix, 2 hours. In all cases, 2 hour time points were used for scale-up and vaccine preparation. Solid arrows indicate TMV-protein conjugate(s), dashed arrows indicate free TMV or free proteins.

*F*. *tularensis* SchuS4 *dnaK* (*FTT1269c*), *ompA* (*FTT0831c*), and *tul4* (*FTT0901*) genes were expressed in *E*. *coli* as N-terminal 6X-His tagged proteins and purified by metal affinity chromatography. The purity of these recombinant proteins was confirmed by SDS-PAGE and western blot analysis using anti-His monoclonal antibodies. Bands of 70kDa, 47kDa and 17kDa confirmed the identities of DnaK, OmpA and Tul4 proteins, respectively (Fig 3). Genetically modified TMV which has a surface exposed Lysine was conjugated with purified DnaK, OmpA and Tul4 proteins of *F*. *tularensis* SchuS4 collectively in a single reaction for generation of TMV-monoconjugate vaccine (Fig 4A) or individually in multiple conjugation reactions to generate TMV-multiconjugate vaccine (Fig 4B, 4C and 4D). The conjugation reaction was anticipated to be complete once the higher molecular weight products were observed on SDS-PAGE gels and the quantity of free recombinant protein in a TMV-protein mixture was less than 10% of the unconjugated controls. Maximum conjugation efficiency was observed after 2 hours of incubation marked by the presence of high molecular weight complexes and disappearance of free proteins.

### Immunization of Mice with TMV-Multiconjugate Vaccine Induces Antibody Responses Capable of Recognizing both Native and Recombinant DnaK, OmpA and Tul4 Proteins

**Fig 5:**
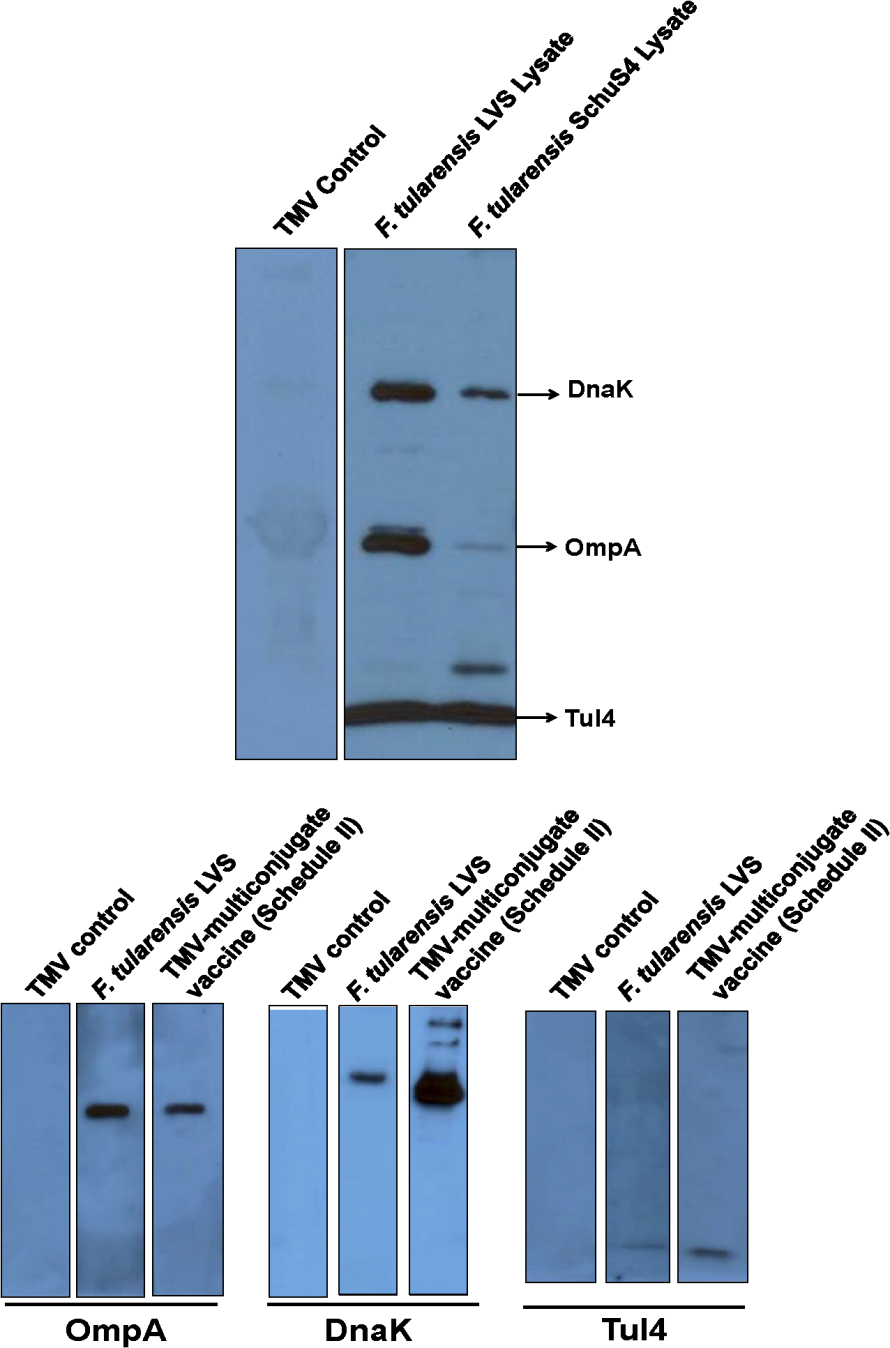
Immunization of Mice with TMV-Multiconjugate Vaccine Induces Antibody Responses Capable of Recognizing both Native and Recombinant OmpA, DnaK and Tul4 Proteins. **(A)** Serum collected on day 28 post-immunization from C57BL/6 mice immunized with TMV-multiconjugate vaccine (Schedule II) was pooled (n = 4) and blotted against *F*. *tularensis* LVS and SchuS4 lysates. **(B)** Pooled serum from C57BL/6 mice (n = 4) immunized either with TMV-multiconjugate vaccine, or 100 CFU of *F*. *tularensis* LVS were collected on day 28 post immunization and blotted against purified recombinant OmpA, DnaK and Tul4 proteins. Sera from mice inoculated with TMV alone were used as controls.

Since purification of recombinant proteins may alter their confirmation or may result in denaturation of immunogenic epitopes, we next investigated if vaccination of mice with TMV-multiconjugate vaccine generates an antibody response capable of recognizing native *Francisella* DnaK, OmpA and Tul4 proteins. Mice immunized with the TMV-multiconjugate vaccine following immunization Schedule II in which mice were boosted by both the i.n. and s.c. routes were bled on day 28 post-immunization. The pooled sera from TMV-multiconjugate vaccine immunized mice specifically recognized DnaK, OmpA and Tul4 proteins in *F*. *tularensis* LVS and SchuS4 lysates indicating that all the antigenic epitopes in immunizing proteins are intact and are capable of recognizing native bacterial proteins (Fig 5A). Conversely, we also investigated if vaccination of mice with live *F*. *tularensis* LVS induces antibody responses against native DnaK, OmpA and Tul4 proteins that can react with the purified recombinant forms of these three proteins. Our results show that sera from mice immunized with live *F*. *tularensis* LVS recognized all three recombinant proteins similar to those observed for sera from mice immunized with TMV-multiconjugate vaccine (Fig 5B).

### Immunization with TMV-Monoconjugate Vaccine Generates Antibody Response Predominated by IgG1 Antibodies

**Fig 6:**
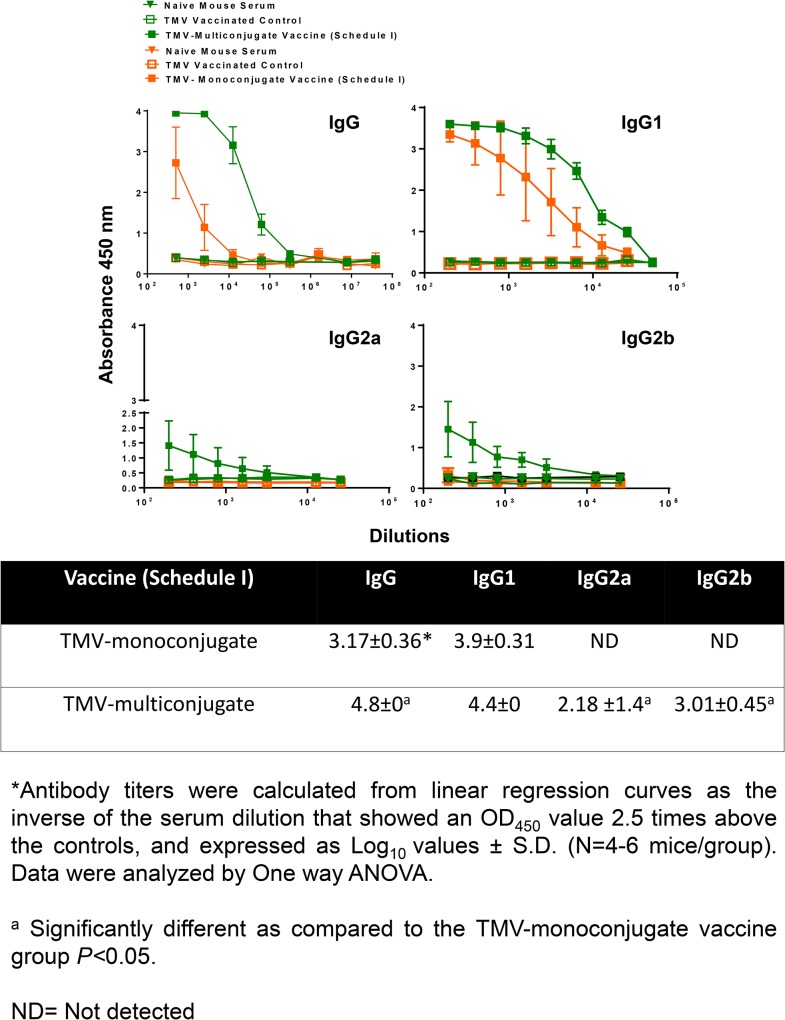
Antibody Responses in Mice Immunized with TMV-Monoconjugate and TMV-Multiconjugate Vaccines using Schedule I of Immunization. *Francisella* specific total IgG, IgG1, IgG2a and IgG2b levels on day 28 in serum samples of C57BL/6 mice immunized with TMV-monoconjugate and TMV-multiconjugate vaccine using Schedule I were determined using an ELISA. Serum samples obtained from naïve mice or those inoculated with TMV alone were used as controls. The data are represented as Mean ±S.D. of absorbance values measured at 450nm. Table shows comparison of antibody titers between groups of mice vaccinated with these vaccine formulations.

**Fig 7:**
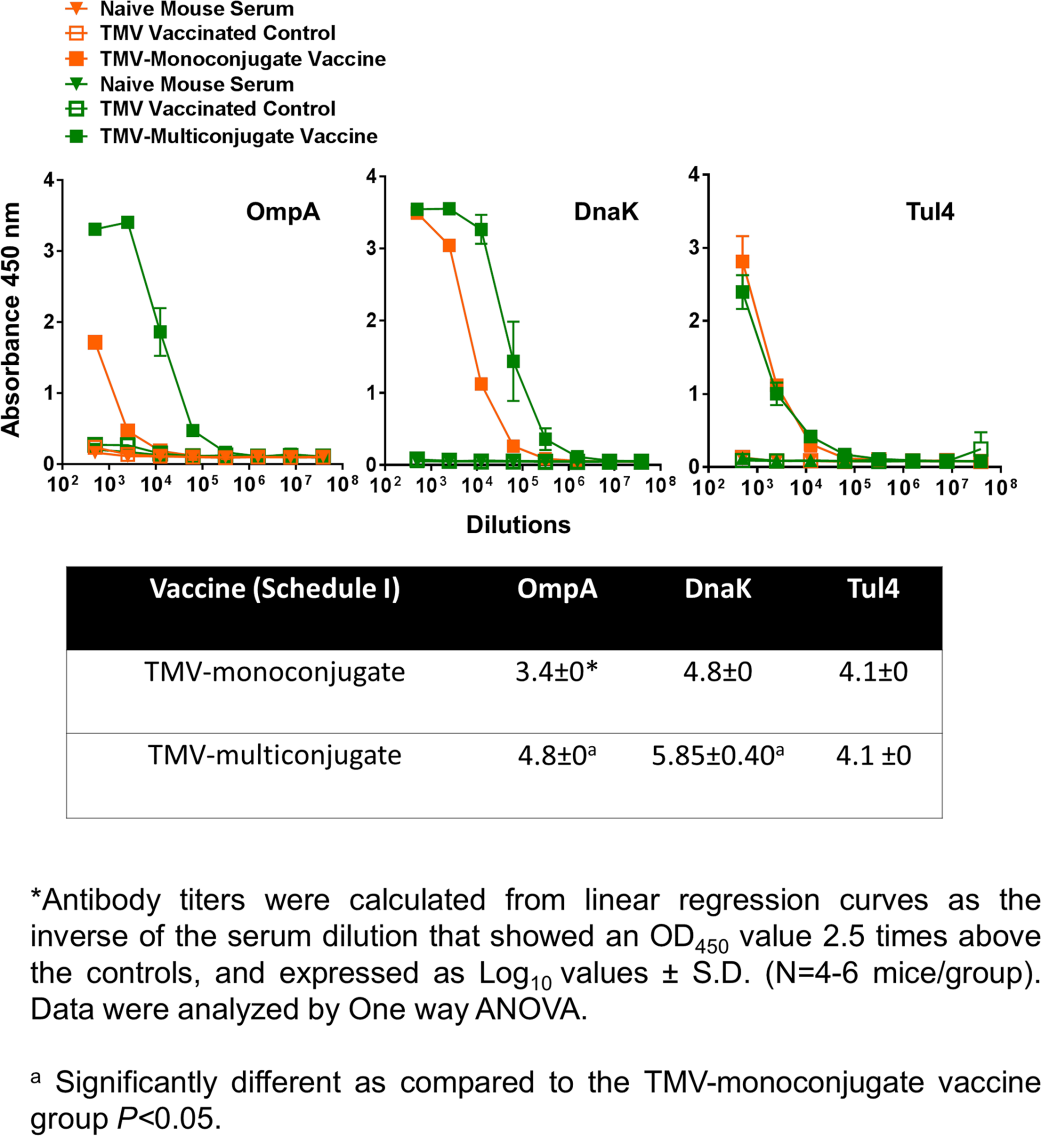
OmpA, DnaK and Tul4 Specific Antibody Responses in Mice Immunized with TMV-Monoconjugate and TMV-Multiconjugate Vaccines using Schedule I of Immunization. *F*. *tularensis* SchuS4 recombinant proteins OmpA, DnaK and Tul4 specific IgG antibody levels on day 28 in serum samples of C57BL/6 mice immunized with TMV-monoconjugate and TMV-multiconjugate vaccine using Schedule I were determined by ELISA. Serum samples obtained from naïve mice or those inoculated with TMV alone were used as controls. The data are represented as Mean ±S.D. of absorbance values measured at 450nm. Table shows comparison of antibody titers between groups of mice vaccinated with these vaccine formulations.

We first investigated the antibody response in mice that received TMV-monoconjugate vaccine formulation in which all the three recombinant proteins were conjugated to a single TMV virion, and received boosters only on day 7 and 14 (Schedule I). Mice were bled on day 28 post-immunization and antibody responses were determined. Higher levels of *Francisella* specific total IgG levels were detected in TMV-monoconjugate vaccinated mice (Fig 6). Determination of IgG isotypes on day 28 post-immunization revealed that mice vaccinated with TMV-monoconjugate vaccine induced higher IgG1 levels. However, very low to undetectable levels of IgG2a and IgG2b antibodies were observed in this group of vaccinated mice (Fig 6). Antigen specific ELISA indicated that antibodies were generated against OmpA, DnaK and Tul4 proteins (Fig 7). Collectively, these results indicated that a weak antibody response predominated by a Th2 biased immune response is generated in mice immunized using Schedule I vaccination regimen with TMV-monoconjugate vaccine formulation.

### Immunization Schedule I with TMV-Multiconjugate Vaccine Generates Stronger Antibody Responses than those Observed with TMV-Monoconjugate Vaccine

We next examined antibody response generated following vaccination with TMV-multi conjugate vaccine in which individual TMV-protein conjugates were blended in a multivalent formulation. The vaccination schedule included booster immunizations on days 7 and 14 post-immunization (Schedule I). The total IgG responses observed on days 14 (not shown) and 28 were much higher than those observed with the TMV-monoconjugate vaccine (Fig 6). IgG isotype profiles were also different in TMV-multiconjugate vaccine than those observed for TMV-monoconjugate vaccine immunized mice. Higher levels of *Francisella* specific IgG2a and IgG2b antibodies were observed in immunized mice. However, as observed for TMV-monoconjugate vaccinated mice, higher levels of IgG1 antibodies were also observed in TMV-multiconjugate vaccinated mice (Fig 6). The levels of OmpA and DnaK specific antibodies were significantly higher in TMV-multiconjugate vaccinated mice than those observed for TMV-monoconjugate vaccinated mice. However, no differences in levels Tul4 specific antibodies were observed in groups of mice vaccinated with these two vaccine formulations (Fig 7). Collectively, these results indicate that TMV-multiconjugate vaccine formulation is a better vaccinogen than the TMV-monoconjugate vaccine however, similar to the latter vaccine formulation, generates an immune response predominated by IgG1 antibodies.

### An Aggressive Immunization with TMV-Multiconjugate Vaccine does not Further Enhance Antibody Response

**Fig 8:**
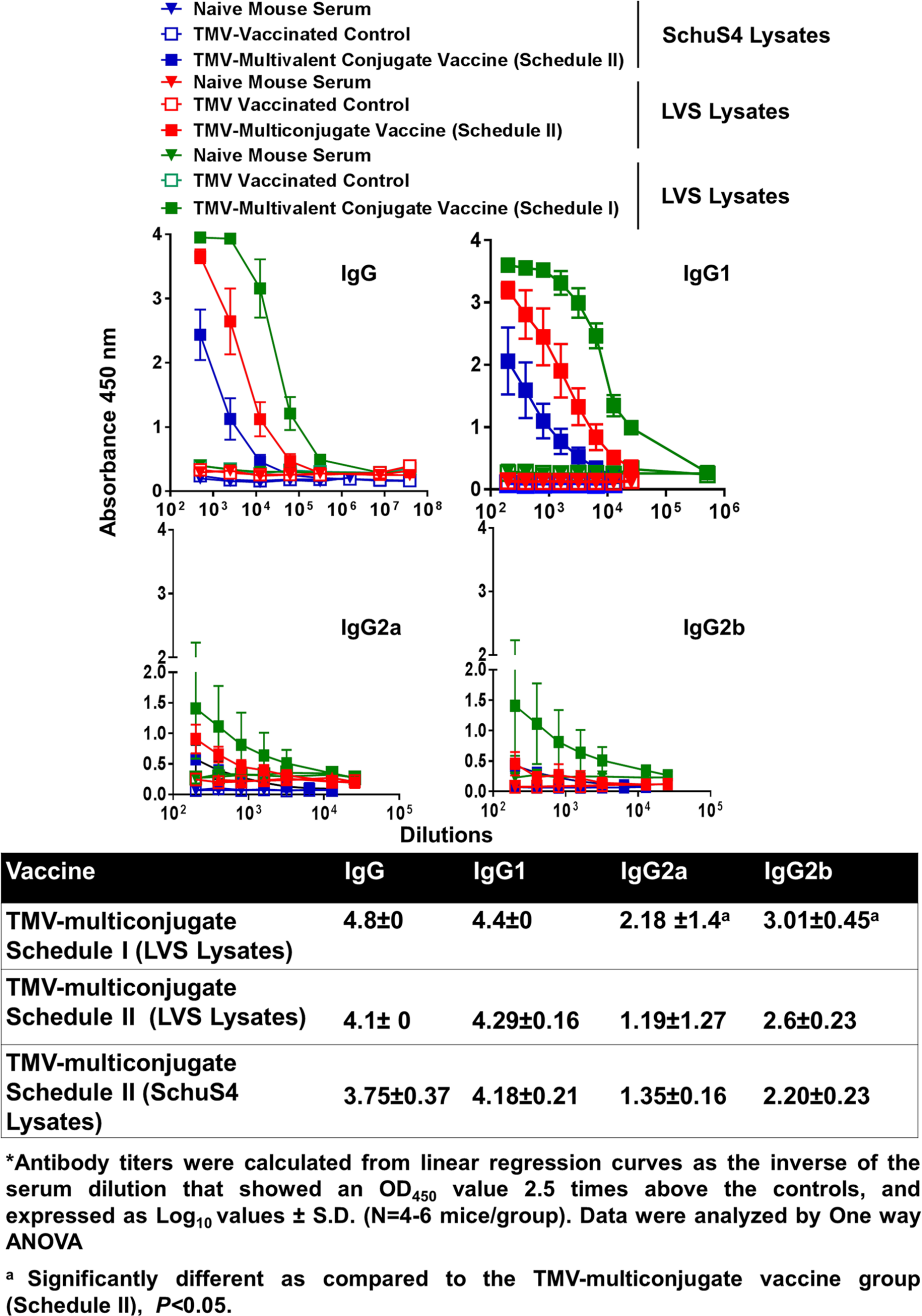
Antibody Responses in Mice Immunized with TMV-Multiconjugate Vaccines using Schedule I and II of Immunization. *Francisella* specific total IgG, IgG1, IgG2a and IgG2b levels on day 28 in serum samples of C57BL/6 mice immunized with TMV-multiconjugate vaccine using Schedule II were determined by ELISA. The plates were coated with *F*. *tularensis* SchuS4 or LVS lysates. Serum samples obtained from naïve mice or those inoculated with TMV alone were used as controls. The data are represented as Mean ±S.D. of absorbance values measured at 450nm. The comparisons are shown with the data obtained from mice immunized with TMV-multiconjugate vaccine using schedule I (shown in Fig 6). Table shows comparison of antibody titers between groups of mice vaccinated with Schedule I and II vaccination regimens.

**Fig 9:**
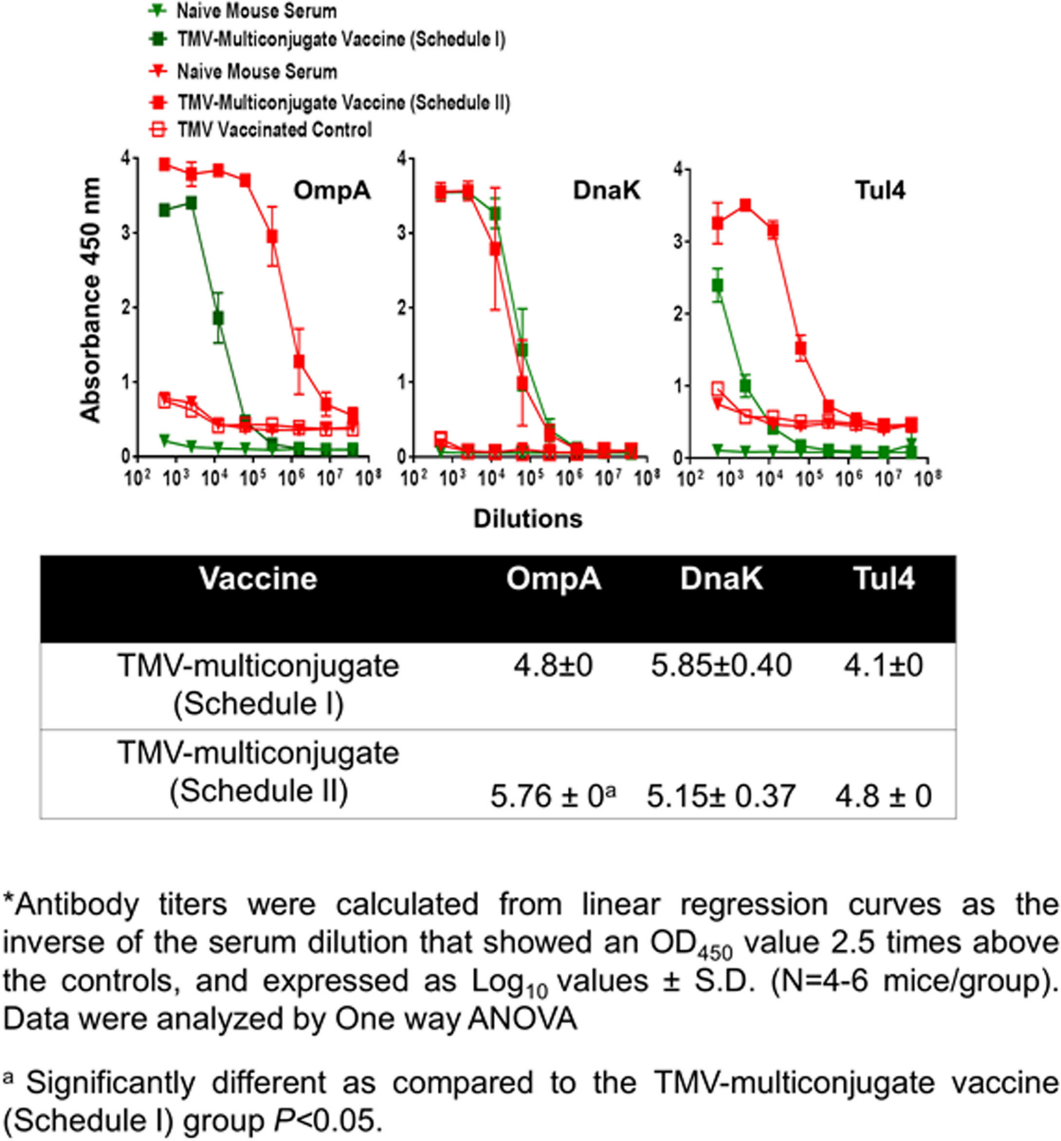
OmpA, DnaK and Tul4 Specific Antibody Responses in Mice Immunized with TMV-Multiconjugate Vaccines using Schedule I and II of Immunization. *F*. *tularensis* SchuS4 recombinant proteins OmpA, DnaK and Tul4 specific IgG, antibody levels on day 28 in serum samples of C57BL/6 mice immunized with TMV-multiconjugate vaccine using Schedule II were determined by ELISA. The plates were coated with recombinant *F*. *tularensis* SchuS4 proteins. Serum samples obtained from naïve mice or those inoculated with TMV alone were used as controls. The data are represented as Mean ±S.D. of absorbance values measured at 450nm. The comparisons are shown with the data obtained from mice immunized with TMV-multiconjugate vaccine using schedule I (shown in Fig 7). Table shows comparison of antibody titers between groups of mice vaccinated with Schedule I and II vaccination regimens.

We further investigated if an aggressive immunization schedule consisting of TMV-multi conjugate formulation administered by both the i.n. and s.c. routes with multiple booster vaccinations (Schedule II) improves the antibody response compared to the other two vaccination strategies. It was observed that the total IgG and IgG1 antibody responses did not differ from mice vaccinated with TMV-multiconjugate vaccine using Schedule I (Fig 8). However, the levels of antibody isotypes IgG2a and IgG2b were in fact significantly higher in mice vaccinated with the TMV-multiconjugate vaccine receiving Schedule I than those receiving Schedule II vaccinations (Fig 8). We further investigated if there are any differences in levels of antibodies generated against native proteins of *F*. *tularensis* SchuS4 and *F*. *tularensis* LVS. No differences in IgG, IgG1, IgG2a and IgG2b antibody levels were observed when ELISAs were performed using *F*. *tularensis* SchuS4 and LVS lysates (Fig 8). These results indicate that similar to the results obtained with western blot analysis, antibodies from TMV-multiconjugate vaccine are equally capable of recognizing native proteins of both *F*. *tularensis* SchuS4 and *F*. *tularensis* LVS. The group of mice receiving Schedule II of TMV-multiconjugate vaccine showed significantly higher titers of OmpA antibodies, while titers of DnaK and Tul4 antibodies were similar to those receiving Schedule I vaccination with TMV-multiconjugate vaccine (Fig 9).

### Protective Efficacy of TMV-Conjugate Vaccine

**Fig 10:**
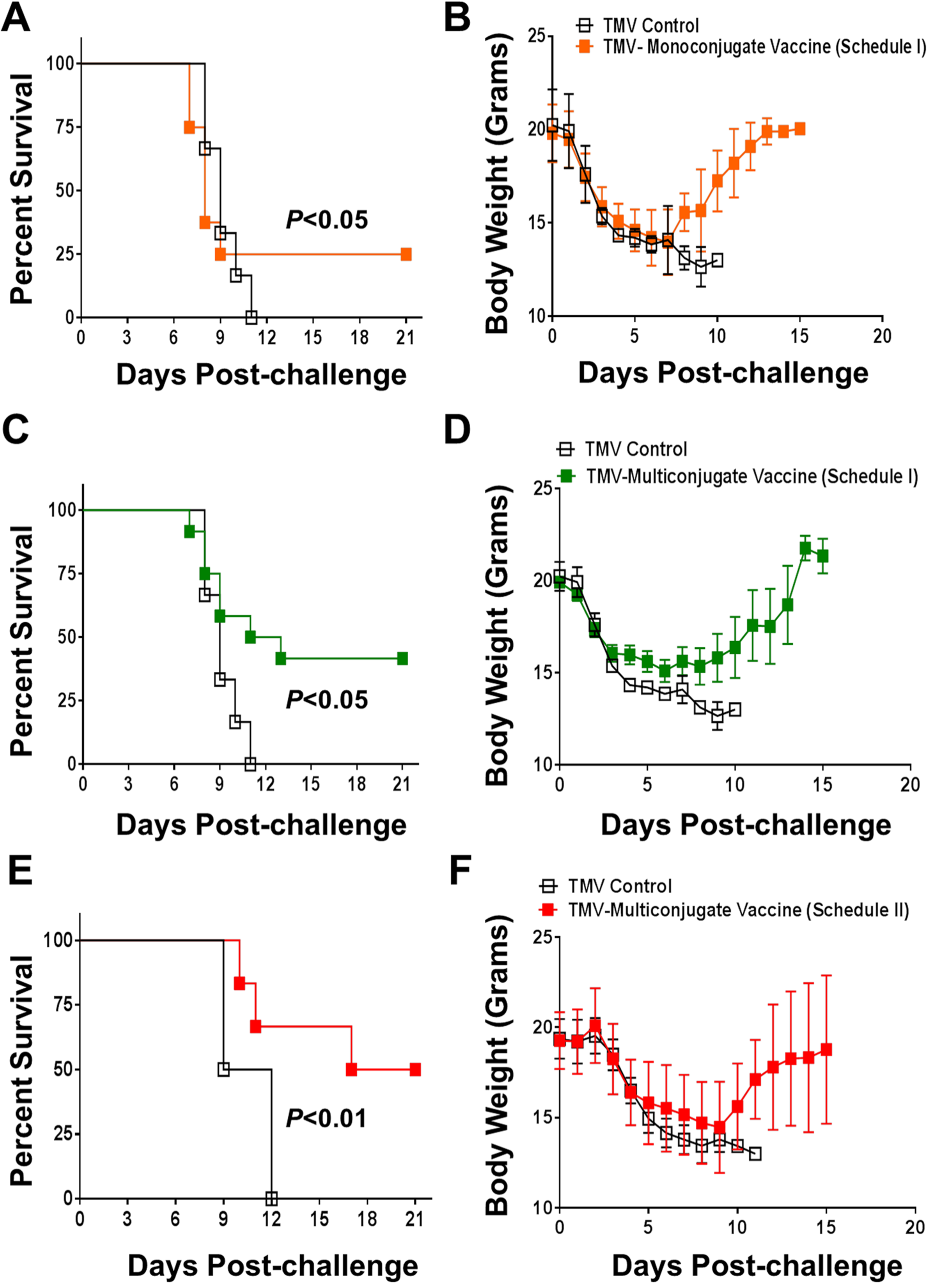
Protective Efficacy of TMV-Conjugate Vaccine. **(A)** C57BL/6 mice (N = 8 per group) immunized with TMV-monoconjugate vaccine; **(C)** with TMV-multiconjugate vaccine (schedule I) or **(E)** with TMV-multiconjugate vaccine (Schedule II) were challenged i.n. with 10xLD_100_ of *F*. *tularensis* LVS on day 28 post-immunization. Mice vaccinated with TMV alone were used as controls. Challenged mice were observed for morbidity and mortality for a period of 21 days post-challenge. The survival results are expressed as Kaplan-Meier survival curves and statistical analysis was performed using Log-rank test. **(B, D and F)** Body weight of the challenged mice at the indicate time points. The data are represented as Mean ± S.D.

We investigated the protective efficacy of the TMV-monoconjugate and TMV-multiconjugate vaccine by vaccinating mice using immunization schedules I and II as described in Fig 2. Mice were immunized with TMV-monoconjugate vaccine using only immunization schedule I; while both schedule I and II were used for TMV-multiconjugate vaccine. Mice were challenged i.n. with 10×LD_100_ dose of *F*. *tularensis* LVS on day 28 post-primary immunization and observed for a period of 21 days for morbidity and mortality. Only 25% of mice immunized with TMV-monoconjugate vaccine survived the challenge while 100% of control mice receiving TMV succumbed to infection by day 11 post-challenge. Measurement of body weights showed an identical pattern of body weight loss between TMV-monoconjugate vaccinated and TMV control groups till day 7 post-challenge after which mice destined for survival started to recover their body weights (Fig 10A and 10B). 40% of mice survived the challenge and started to regain their body weights by day 8 post-challenge in the group of mice that received TMV-multiconjugate vaccine using Schedule I vaccination regimen. All the control mice progressively continued to lose weight and succumbed to challenge (Fig 10C and 10D). The pattern of survival and body weight loss of mice receiving TMV-multiconjugate vaccine with Schedule II was similar to that observed for mice vaccinated with Schedule I regimen, however 50% of the vaccinated mice survived the challenge (Fig 10E and 10F). Additional booster vaccinations by i.n. and s.c. routes in Schedule II only slightly improved the level of protection in this group of vaccinated mice. Collectively, these results demonstrated that *Francisella* proteins conjugated to TMV when used as vaccine induce protective immune response in mice. These results also indicated that TMV-monoconjugate vaccine in which all the three recombinant proteins OmpA, DnaK and Tul4 of *F*. *tularensis* conjugated to a single TMV virion serves as a poor vaccinogen. On the other hand, the vaccine formulation that contains a multivalent blend of all the three proteins conjugated individually to TMV induces a superior protective immune response that can marginally be improved further by increasing number of booster vaccinations by s.c. route.

## Discussion

The possibility of using *F*. *tularensis* as a bioterror agent has renewed attention towards *F*. *tularensis* research and to develop a licensable vaccine for effective prevention of tularemia. The tularemia vaccine development research has largely been focused on development of live attenuated or inactivated tularemia vaccines. However, concerns about their efficacy and safety have halted the progress. A recent study confirms this notion and reported a variant of *F*. *tularensis* LVS which is 100 times lethal than the standard ATCC strain indicating that, as feared, *F*. *tularensis* LVS may revert back to its virulent form [46]. Recombinant subunit vaccines obviously have potential safety advantages over inactivated or live attenuated vaccines.

Ongoing studies for subunit vaccine development have identified a number of *F*. *tularensis* antigens that are capable of inducing a partial protective immune response [47,48,49,50,51]. The possible explanation for limited protective efficacy of these subunit vaccines could be that a single protein or combinations of proteins used in vaccine formulations were not sufficient to induce an efficient protective immune response. Another shortcoming appeared to be the availability of a suitable platform for simultaneous delivery of antigens in a consistent fashion. It has been shown that the protective efficacy improves when multiple antigens are used in vaccine formulations [52,53,54]. However, the biggest challenge thus far in the development of multivalent subunit vaccines has been the availability of suitable approaches for consistent preparation and efficient delivery of multiple antigens through mucosal route.

The goal of this study was to explore vaccine potential of a multivalent subunit vaccine against tularemia using an efficient TMV based delivery platform. The premise behind utilizing a novel TMV-conjugated vaccination strategy is based on the proven efficacy of TMV vaccines in stimulating robust immune response without the requirement of an additional adjuvant [29]. In order to provide a proof-of-concept and feasibility of TMV as a carrier for *F*. *tularensis* proteins, we used purified recombinant proteins OmpA, DnaK and Tul4 from *F*. *tularensis* SchuS4 for conjugation studies and determined the vaccine potential of TMV-*F*. *tularensis* protein conjugates. Two different vaccine formulations consisting either of all three *F*. *tularensis* proteins conjugated to a single TMV virion (TMV-monoconjugate vaccine); or a mixture consisting of each protein individually conjugated to TMV (TMV-multiconjugate vaccine) were used. Both the vaccine formulations resulted in generation of antibodies against all three recombinant proteins. Most importantly, these antibodies recognized native *Francisella* as well as recombinant proteins. These results indicate that the purification or conjugation procedures did not alter conformation of the epitopes recognized in native OmpA, DnaK or the Tul4 proteins.

When immune responses were compared between mice receiving TMV-monoconjugate and TMV-multiconjugate vaccine using a similar vaccination regimen (Schedule I), a weaker antibody response was observed in mice vaccinated with monoconjugate formulation. These results indicate that conjugating all proteins to a single TMV virion is not an ideal approach for development of a TMV-based tularemia vaccine. The poor antibody response could be due to preferential conjugation of one of the three proteins in the conjugation mix or due to antigenic competition. The antibody responses observed against OmpA and DnaK proteins does support this notion. It was observed that TMV-monoconjugate vaccinated mice induced antibody response against Tul4 protein were similar to those observed for TMV-multiconjugate vaccine formulations however, the response against OmpA and DnaK were significantly lower than that observed for the latter vaccine formulation (Fig 7). The binding capacity of each of these proteins to TMV was not determined in the present study. However, these results do point to the fact that Tul4 due to its smaller size may have a preferential binding to the surface of TMV than OmpA or DnaK proteins. Moreover, the immune response was predominated by IgG1 antibodies and no IgG2a or IgG2b responses were observed in mice vaccinated with TMV-monoconjugate vaccine. These results indicate generation of a predominantly Th2 biased immune response in this group of vaccinated mice. Contrary to what was observed for TMV-monoconjugate vaccinated mice, the TMV-multiconjugate vaccinated mice mounted a very strong *Francisella* specific total IgG response and the titers went up from days 14 to 28 post-immunization (not shown). Although, IgG1 was the most predominant antibody isotype, higher levels of IgG2a and IgG2b antibodies were also detected in this group of immunized mice. It is noteworthy that both the IgG2a and IgG2b antibodies have been shown to be protective against *F*. *tularensis* infection [55,56]. Collectively, these results indicate that conjugating each protein individually to TMV and then blending them in equimolar concentration to generate a multiconjugate composition is an ideal approach for the development of a TMV-based tularemia vaccine.

We further investigated if an enhanced immune response can be generated following an aggressive vaccination regimen with the TMV-multiconjugate vaccine. We administered booster vaccinations by alternating i.n. and s.c. routes. The intent was to induce potent systemic as well as local mucosal immune response by administering vaccine by both i.n. and s.c. routes (Schedule II). However, the immune responses did not differ from those observed in mice receiving only two booster vaccines (Schedule I). We speculate that the failure to observe further amplification following an aggressive vaccination could be due to an excessive antigenic overload. When vaccinated mice were challenged intranasally with 10LD_100_ dose (1x10^5^ CFU) of *Ft* LVS, corresponding to the weak antibody response only 25% mice were protected with TMV-monoconjugate vaccine formulation. However, with multiconjugate formulation the protection levels were nearly 40% and an aggressive immunization marginally improved the protection to 50%. Collectively, these results demonstrate that TMV can be used as a carrier for effective delivery of multiple *F*. *tularensis* antigens. The TMV-conjugate vaccine is safe and multiple doses can be administered in mice without any adverse reactions and immunization with *F*. *tularensis* antigens conjugated individually and blended in a multivalent composition induce a more potent immune response than the formulation in which all of the three proteins are conjugated to a single TMV virion. Most importantly immunization with TMV-conjugated *F*. *tularensis* proteins can protect mice against lethal doses of *F*. *tularensis* LVS.

In conclusion, this study provides a proof-of-concept that TMV can serve as a suitable platform for simultaneous delivery of multiple protective antigens of *F*. *tularensis*. Future studies to improve the level of protection would require generation of TMV-multiconjugate vaccine by incorporating additional immunoprotective antigens of *F*. *tularensis* and inclusion of suitable adjuvant(s) to generate potent humoral and cell-mediated immune responses and induce long-lasting immunity against tularemia caused by *F*. *tularensis* SchuS4 strain.
